# Anatomical Study and CT Scan of the Scleral Ring in the Little Owl (*Athene noctua*)

**DOI:** 10.1002/vms3.70845

**Published:** 2026-02-15

**Authors:** Omid Zehtabvar, Majid Masoudifard, Hesameddin Akbarein, Arman Shahbazi, Setayesh Karimi, Sara Binesh, Elnaz Khalilzadeh

**Affiliations:** ^1^ Anatomy Sector, Department of Basic Sciences, Faculty of Veterinary Medicine University of Tehran Tehran Iran; ^2^ Department of Surgery and Radiology, Faculty of Veterinary Medicine University of Tehran Tehran Iran; ^3^ Department of Food Hygiene and Quality Control, Faculty of Veterinary Medicine University of Tehran Tehran Iran; ^4^ Faculty of Veterinary Medicine University of Tehran Tehran Iran; ^5^ Faculty of Veterinary Medicine Islamic Azad University, Science and Research Branch Tehran Iran

**Keywords:** anatomy, CT scan, little owl, ossicle, scleral ring

## Abstract

*Athene noctua*, commonly known as the little owl, thrives across the warmer climates of Europe, Asia and North Africa. One of the anatomical features of birds is the presence of a bony scleral ring in the eye. In avians, this configuration comprises ossicles that are affixed together in diminutive plates and are not articulated to other components of the skeleton. The morphology, number, development and location of the scleral ring vary among different vertebrate groups. The objective of this research is to furnish a comprehensive elucidation of the morphology of the scleral ring in the *A. noctua* predicated on CT scan results and anatomical examination. The overall shape of the scleral ring, the number and shape of the ossicles and their positioning and extensions are notable features that can be used for classification purposes. The study population comprised 10 adult owls (five male and five female). Micro‐CT scan, CT scan, ultrasound, radiography and morphometric analysis were used for these owls. The results indicated that the scleral ring in the owls consisted of 15 ossicles, arranged in quadrilateral and rectangular shapes. In one sample, the right eye ring of the owl contained 16 ossicles, which was considered an exceptional feature. The ring consisted of two parts: an anterior tubular section and a posterior conical section. Morphometric analysis showed significant differences between male and female owls in various measurements.

**Summary:**

- *Ossicle Composition*: The scleral ring in *Athene noctua* predominantly consists of 15 quadrilateral ossicles, with a rare anatomical variation of 16 ossicles observed unilaterally in one specimen.
- *Structural Morphology*: The ring displays a distinct bipartite architecture, featuring an anterior tubular segment with a near‐circular cross‐section and a posterior funnel‐shaped segment with oval cross‐section.
- *Sexual Dimorphism*: Morphometric analysis revealed statistically significant (*p* < 0.05) larger ocular dimensions in female specimens compared to males most measured parameters.
- *Absence of Sesamoid Bone*: Unlike other strigiform species, no sesamoid ossification or tubercular structures were observed adjacent to the scleral ring.
- *Taxonomic and Clinical Relevance*: These findings provide (1) diagnostic markers for ocular trauma assessment and (2) potential phylogenetic discriminators within Strigiformes.

## Graphical abstract

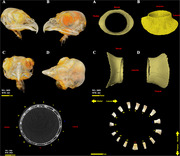

## 1 Introduction

Little owl (*Athene noctua*) is a species of owl from the genus *Athene*. This bird lives in the warm regions of Europe, Asia and North Africa (Greenoak 1997; Jobling 2010). The body length of this bird is 21–23 cm and the wingspan is 54–58 cm (Varasteh et al. 2018). The eyeball in birds is similar to the eyeball of mammals in terms of overall structure, but in birds, the ratio of eye size to head size is much larger than mammals (King and McLelland 1984). The eyeball in diurnal birds is large and is completely placed in the orbit, thus protecting it from external damage. Instead, in many crepuscular birds such as owls, the orbit is shallow and provides little protection to the eyeball; in these birds, this task is mostly on the scleral ring (König et al. 2016). The size and weight of the eyeball of birds are proportional to their function. In most bird species, the relative weight of the eyeball is higher than mammals; for example, the weight of the eyeball is 7%–8.5% of the head weight in chickens, 17%–21.5% in pigeons and birds of prey, and more than 22%–32% in owls. This ratio is much higher than that of humans (about 1%) (König et al. 2016). The eyeball in birds is not spherical, and the anterior part of the eyeball, covered by the cornea, has a less curvature than the posterior part of the eyeball. As a result, it creates a shallow and plate‐like body (König et al. 2016). The bones inside the sclera are arranged as groups of plates or ossicles that form the scleral ring, and these ossicles are dermal bones (Franz‐Odendaal 2008). The bony ring of the sclera provides mechanical strength to the concave annular part of the eyeball. The scleral is made up of 10–18 (usually 15) individual ossicles that are arranged together like fish scales (König et al. 2016). Two principal functions have been posited for the scleral ring: one function is to inhibit alterations in the shape of the eyeball during aerial flight or aquatic diving, while the second function is to facilitate the operation of the ciliary muscles and contribute to the alignment of visual acuity. However, the precise roles of each individual ossicle remain a subject of ongoing scholarly debate (Lima et al. 2017). The scleral ring delineates an intermediary region between the anterior segment of the ocular globe, which accommodates the cornea, and the posterior segment, which contains substantial volume. The interstitial region is elongated across all species of owls, imparting an elongated morphology to the anterior section of the eyeball; consequently, it is commonly asserted that the eyes of owls exhibit a tubular characteristic (King and McLelland 1984). The presence of the scleral ring is documented in various reptilian species. In reptiles, the quantity of ossicles is notably less than that observed in avian species (Franz‐Odendaal 2006). In some species of reptiles, such as crocodiles and some snakes, only the cartilaginous structure is present in the eye (King and McLelland 1984). The larger scleral ring belongs to fast animals, and tubular scleral rings are more common in the eyes of owls (Burton 2008). The ossicles forming the scleral ring are involved in the formation of eye length. This characteristic is noticeable in owls, so that the length of the scleral ossicle in owls is twice its width, and the largest scleral ring has been seen in owl species. In the scleral ring, the ossicles are numbered sequentially, beginning with a prominent plus ossicle (designated as ossicle number 1) located in the ventral region. The numbering then proceeds along the lateral portion of the ring. The ossicles are numbered counterclockwise in the left eye.

Notably, certain ossicles—referred to as excellent ossicles—play a key role in modulating the pattern of the ring's covering. Two types of key ossicles (excellent ossicles) are plus and minus ossicles. If the articular surface of an ossicle covers both of its lateral ossicles, it is called a positive ossicle, and if its articular surfaces are covered by the lateral ossicles, it is called a negative ossicle. If one surface of an ossicle is covered by the lateral ossicle and the other surface causes the lateral ossicle to be covered, it is called an interlocking ossicle (Fischer and Schoenemann 2019). Scleral rings in avian species are classified into two categories, A and B, contingent upon the quantity of key ossicles present. Category A comprises four key ossicles, whereas Category B contains two key ossicles; notably, Category B is observed in owls and predatory birds. The quantity of ossicles in the right and left ocular structures may exhibit variation (Fischer and Schoenemann 2019).

The overall anatomical configuration of the scleral ring in Strigiformes remains consistent, and variations in both morphology and function among the scleral rings of various owl species have not been documented. A distinctive anatomical feature among owls is the presence of a scleral sesamoid bone (a small, variably shaped structure) that is located adjacent to the scleral ring (Lima et al. 2017). This ossification displays significant morphological diversity across species. In specific instances, such as in *Asio stygius*, this structure resembles a discrete osseous formation positioned separately alongside the scleral ring, whereas in another species, namely *Speotyto cunicularia*, this structure is characterised as a tubercle (Lima et al. 2017). Studies on the structural characteristics of birds' eyes have been conducted using various diagnostic imaging methods, and in some of them, the eyes of different species of owls have also been studied. One of the species investigated in the mentioned studies is *Asio otus* (Zehtabvar et al. 2022).

It should be noted that not many studies have been conducted on the scleral ring in the little owl. Also, considering the general injuries that may be caused to the skull and eyeball, having detailed information about the natural structure of the scleral ring can be useful in diagnosing injuries and fractures. The goal of this study is to provide an exact description of the anatomy of the scleral ring of *A. noctua* based on the findings of digital radiography, CT scan and anatomy. The scleral ring's morphological characteristics—including its general configuration, ossicle count and morphology, and spatial arrangement of components—serve as taxonomically informative traits for classification purposes, and considering the general structure of this ring in the little owl and the differences it has with other species, this factor can be used in the future for a more detailed classification.

## 2 Materials and Methods

The species examined in this study was the little owl (*A. noctua*). The study population included five live adult male owls (210 ± 10.22 g) and five live adult female owls (225 ± 11.98 g), which were treated for movement problems in a veterinary clinic. At first, the owls were anesthetised by intramuscular injection of ketamine (10 mg/kg) and xylazine (2 mg/kg) (Carpenter and Marion 2017). The micro‐CT scan studies were performed on owl cadavers collected from veterinary clinics across the city.

### 2.1 CT Scan Studies

In this study, Somatom Spirit model 2 CT scan device manufactured by Siemens was used (*n* = 10). Morphometric measurements were performed using the Syngo‐MMWP‐VE40A software available in the CT scan. CT scan technical factors are as follows:

Rotation time: 1 s, reconstruction interval: 0.5–1 mm, slice thickness: 1 mm, pitch: 1, x‐ray tube current: 130 mA s and x‐ray tube potential: 120 kV.

### 2.2 Radiographic Studies

In this study, the Kodak DirectView CR 850 digital radiography device located in the faculty of veterinary medicine (University of Tehran, Iran) will be used (*n* = 10). x‐Ray images were prepared in dorsoventral and lateral views, as well as factors including 4 mA s and 45 kVp.

### 2.3 Micro‐CT Scan Studies

**FIGURE 1:**
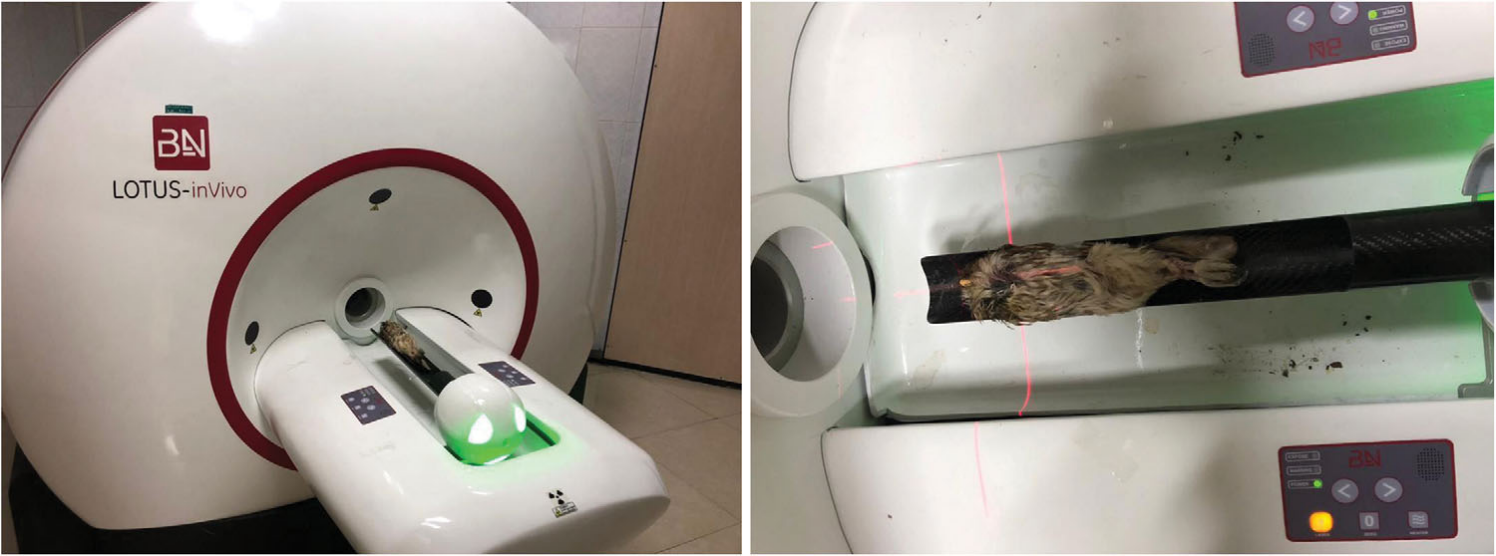
The positioning of the little owl for micro‐CT scan imaging.

These studies were performed in the preclinical laboratory of Tehran University of Medical Sciences using the LOTUS‐inVivo device. The owl samples were sent frozen to the mentioned centre (*n* = 3 male + 2 female). The sample was placed inside the micro‐CT machine and two‐dimensional (2D) and three‐dimensional (3D) images of the owl's head were prepared. In this study, the settings of the device were as follows: x‐ray tube voltage: 70 kV, frame exposure time: 1 s by 3 magnification and slice thicknesses of reconstructed images: 25 µm (Figure 1).

### 2.4 Ultrasonographic Study

For efficient cranial and ocular ultrasonography, the ultrasonography machine and Linear Multi‐Frequency Probe were preferred (GE Voluson 730, 3–11 MHz). Sagittal sonograms were taken from the eyes.

### 2.5 Gross Anatomical Dissection

For this section of the study, the heads of five owls that died due to various reasons and were referred to the Anatomy Department of the Faculty of Veterinary Medicine, University of Tehran were collected and separated (*n* = 3 male + 2 female). The bone structures of the skull were separated for 8 days using the insect method (*Tenebrio molitor*). After cleaning the bones, the specimens were subsequently relocated to an Olympus SZX12 stereo microscope for comprehensive analysis and image preparation.

### 2.6 Morphometric Studies

Morphometric measurements were performed using the Syngo MMWP VE40A software available in the CT scan device. The independent *t*‐test and the paired *t*‐test were employed to conduct a comprehensive analysis of the data. *p* ≤ 0.05 was considered significant. The method of measuring different parameters in this study is shown in Table 1 (Masoudifard et al. 2025). Measurements were performed on 2D CT scan images (although the images shown in Table 1 are in some cases on 3D reconstructed images for better explanation).

**TABLE 1:** Morphometric measurements performed in this study.

Description	Parameter	Visual description
Transverse (horizontal) diameter of the anterior segment of the scleral ring. Measurements were acquired from three‐dimensional computed tomography scan images.	Anterior diameter of scleral ring	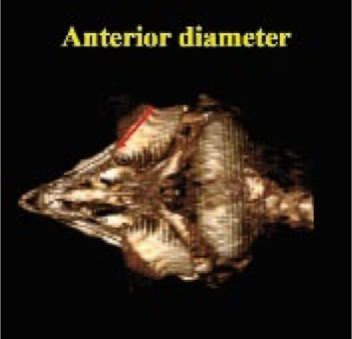
Transverse (horizontal) diameter of the posterior segment of the scleral ring. Measurements were acquired from three‐dimensional computed tomography scan images.	Posterior diameter of scleral ring	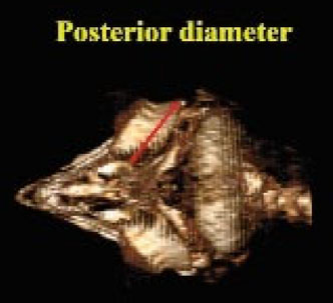
The linear separation along the horizontal axis between the anterior and posterior boundaries of the scleral ring. Measurements were acquired from three‐dimensional computed tomography scan images.	Length of scleral ring	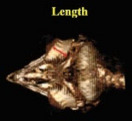
The linear extent in the horizontal plane from the anterior to the posterior margin of the ocular globe. Quantitative assessments were performed utilizing two‐dimensional coronal computed tomography imaging.	Anterior–posterior diameter of eyeball	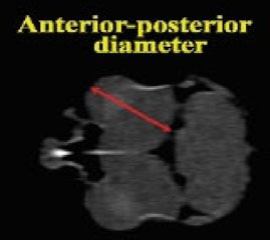
The optic nerve was discerned within the caudomedial region of the ocular globe. Quantitative assessments were performed utilizing two‐dimensional coronal micro–computed tomography imaging.	Optic nerve length	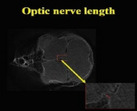
The optic nerve was discerned within the caudomedial region of the ocular globe. Quantitative assessments were performed utilizing two‐dimensional coronal micro–computed tomography imaging.	Optic nerve sheath diameter	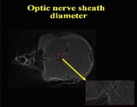
The anatomical positioning of the *Pecten* structure was evaluated through the utilization of sagittal ultrasound imaging. Quantitative assessments were conducted utilizing the sagittal ultrasonogram.	Height of *Pecten*	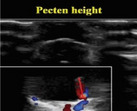

## 3 Results

### 3.1 Results of Radiographic Images

**FIGURE 2:**
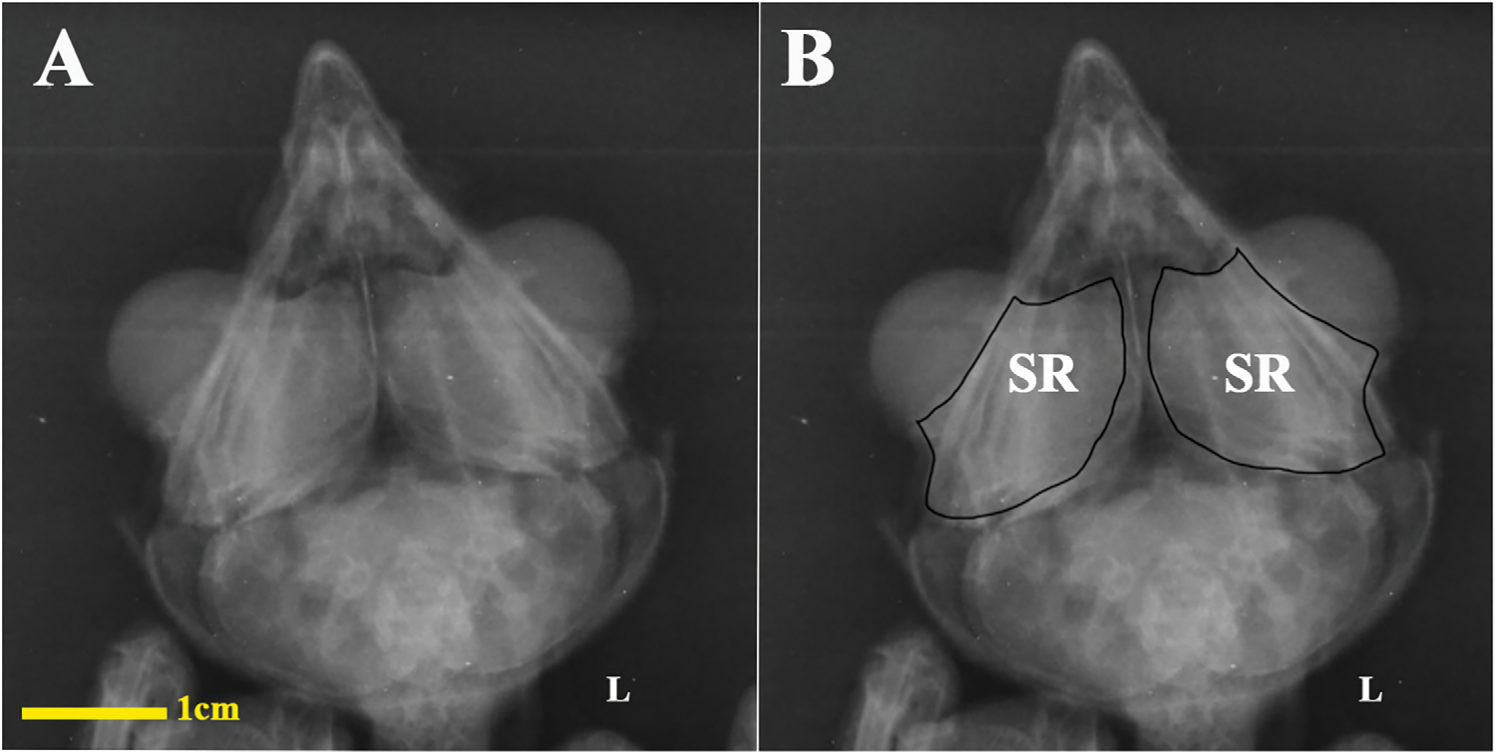
Radiographic image of the head in a little owl (*Athene noctua*), dorsal‐ventral view. SR: scleral ring.

In the dorsoventral and lateral images, the scleral ring exhibiting bone opacity was distinctly observable. In the lateral view, due to the overlap of the skull structures, it was difficult to fully examine the scleral ring, and the dorsoventral view showed this structure more clearly and separately. The lack of connection to other parts of the skull, especially in the dorsoventral view, was completely observed. Although it was not possible to separate and count the ossicles that make up this structure in the radiographic images, the location, normal opacity of the bone and to some extent the integrity of this structure could be determined in the radiographic images, although the border of the ossicles was somewhat clear (Figure 2).

### 3.2 Results of Anatomical Examination in CT Scan Images and Bone Samples

**FIGURE 3:**
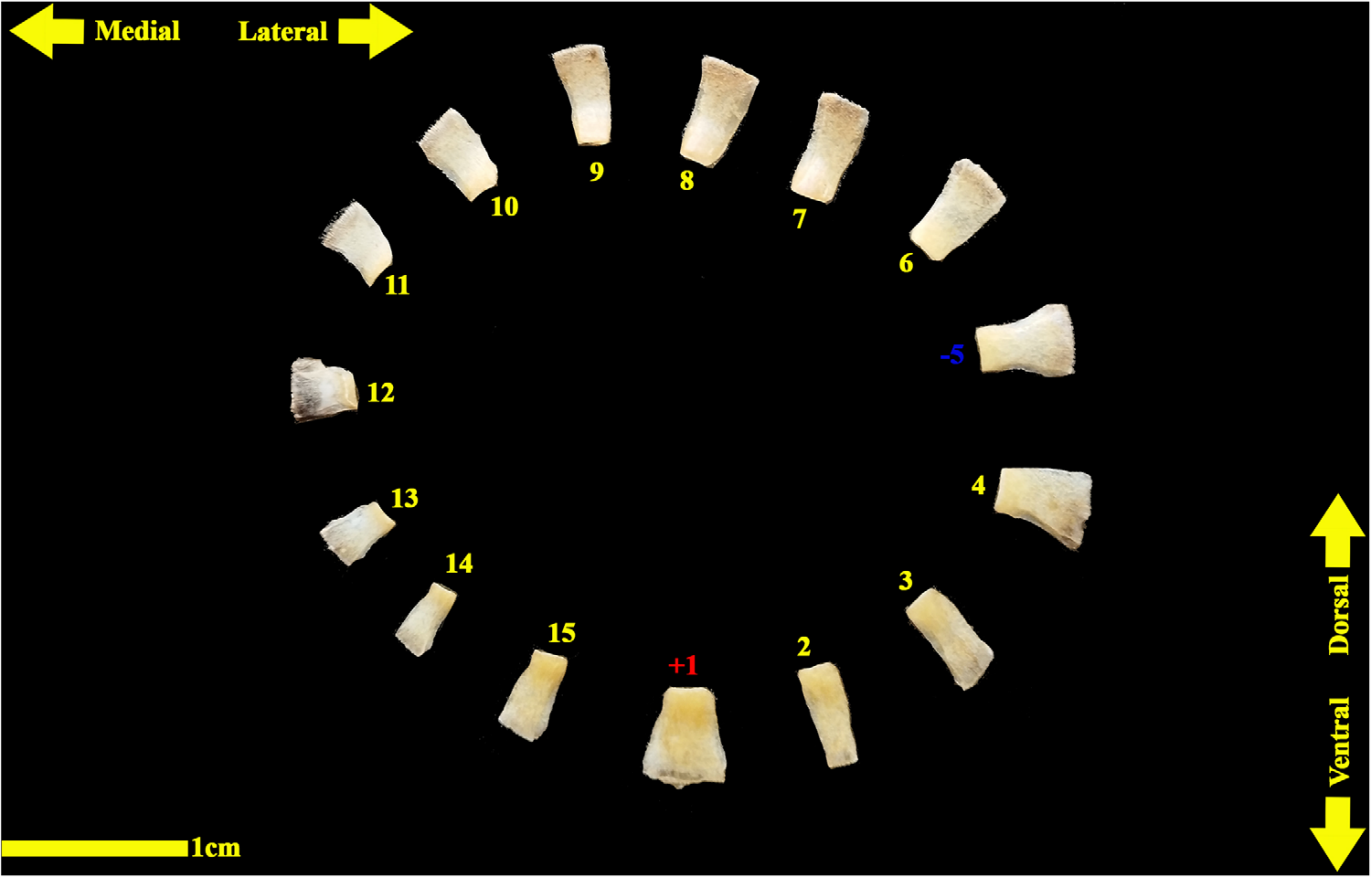
Anterior view of a sample of isolated ossicles of the left eye scleral ring in a little owl.

**FIGURE 4:**
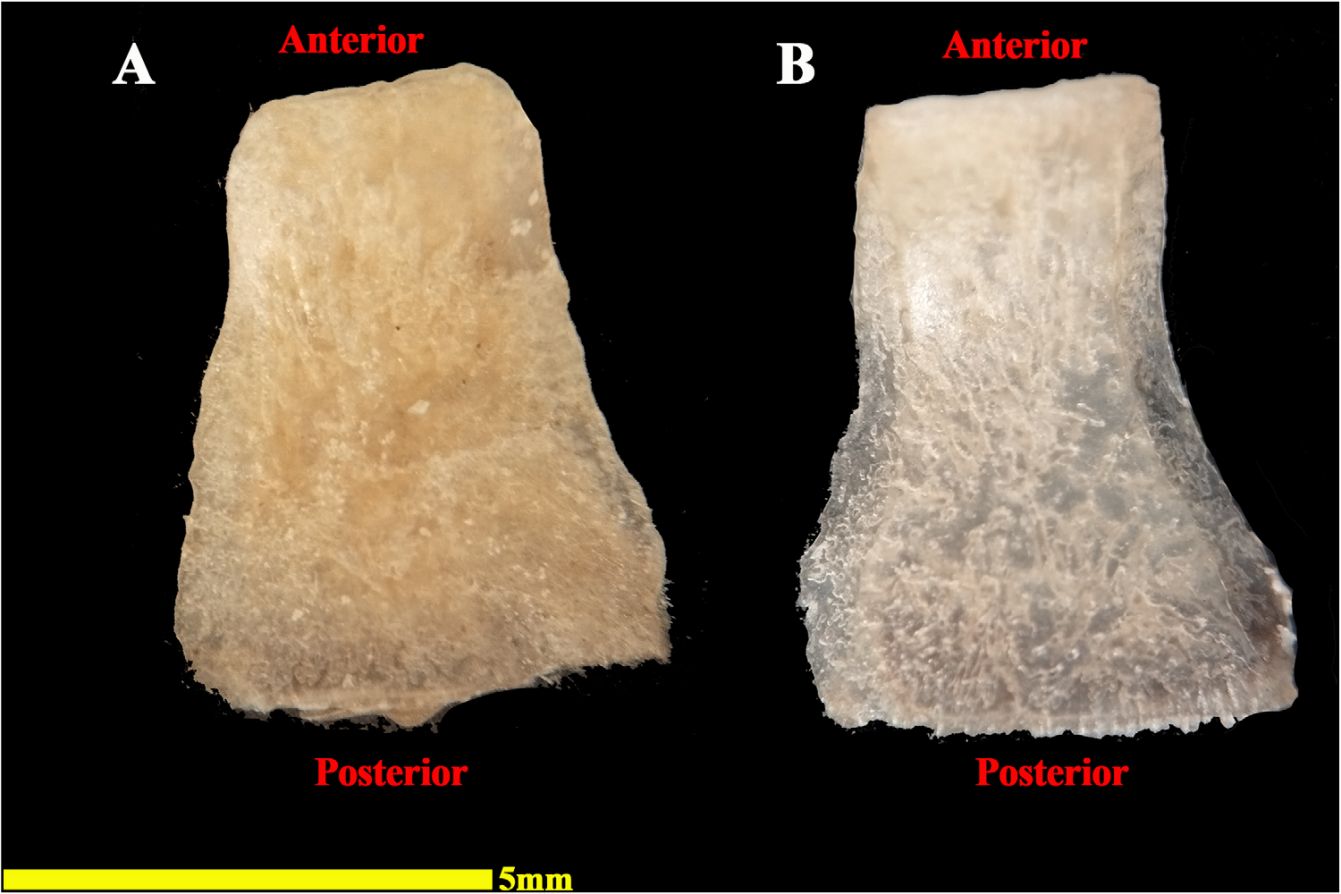
A sample of isolated ossicles of the scleral ring of the left eye in a little owl (outer surface). (A) +1st ossicle and (B) −5th ossicle.

**FIGURE 5:**
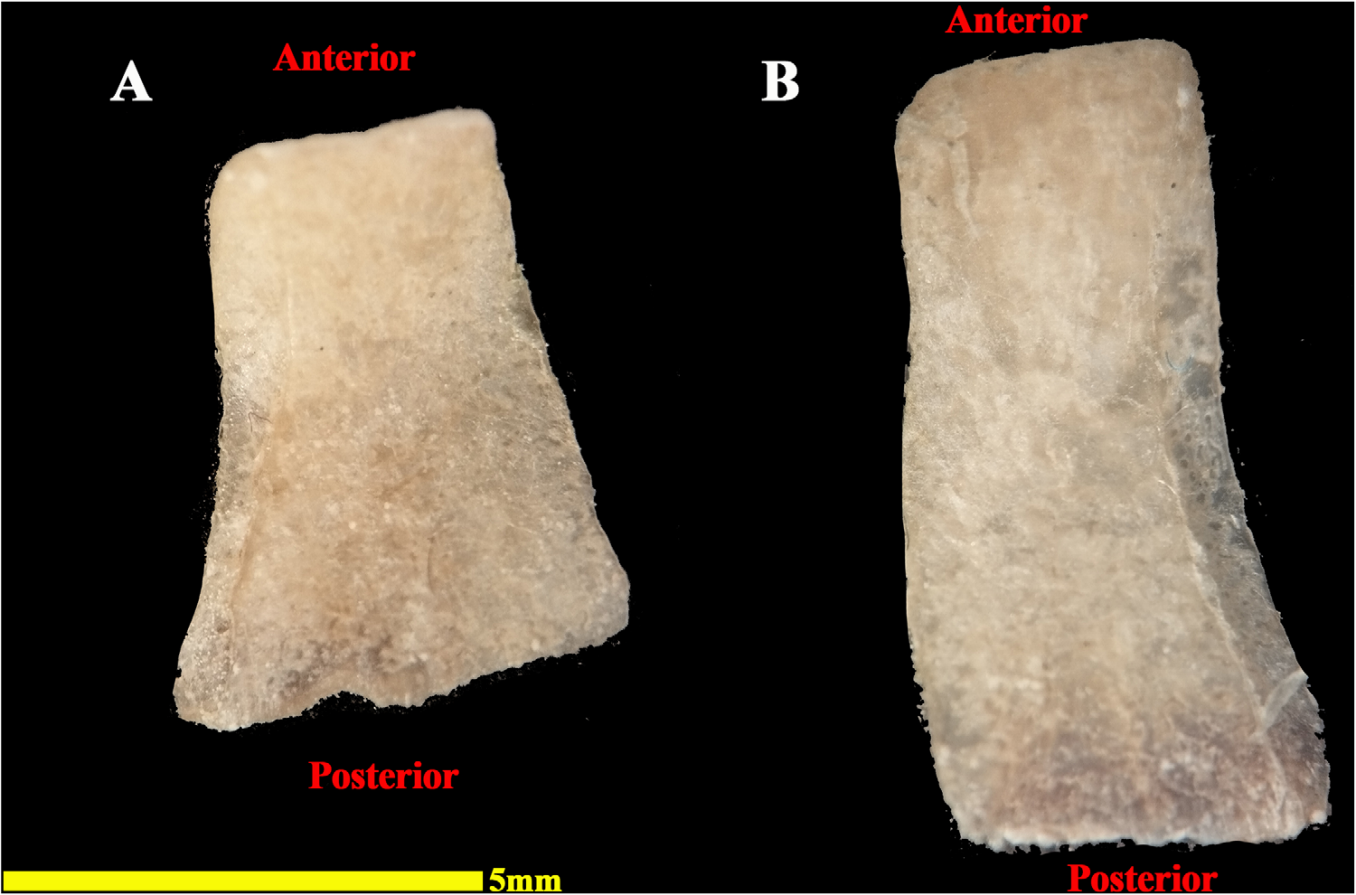
Samples of isolated ossicles of the scleral ring of the left eye in a little owl (*Athene noctua*). (A) 4th ossicle and (B) 15th ossicle.

**FIGURE 6:**
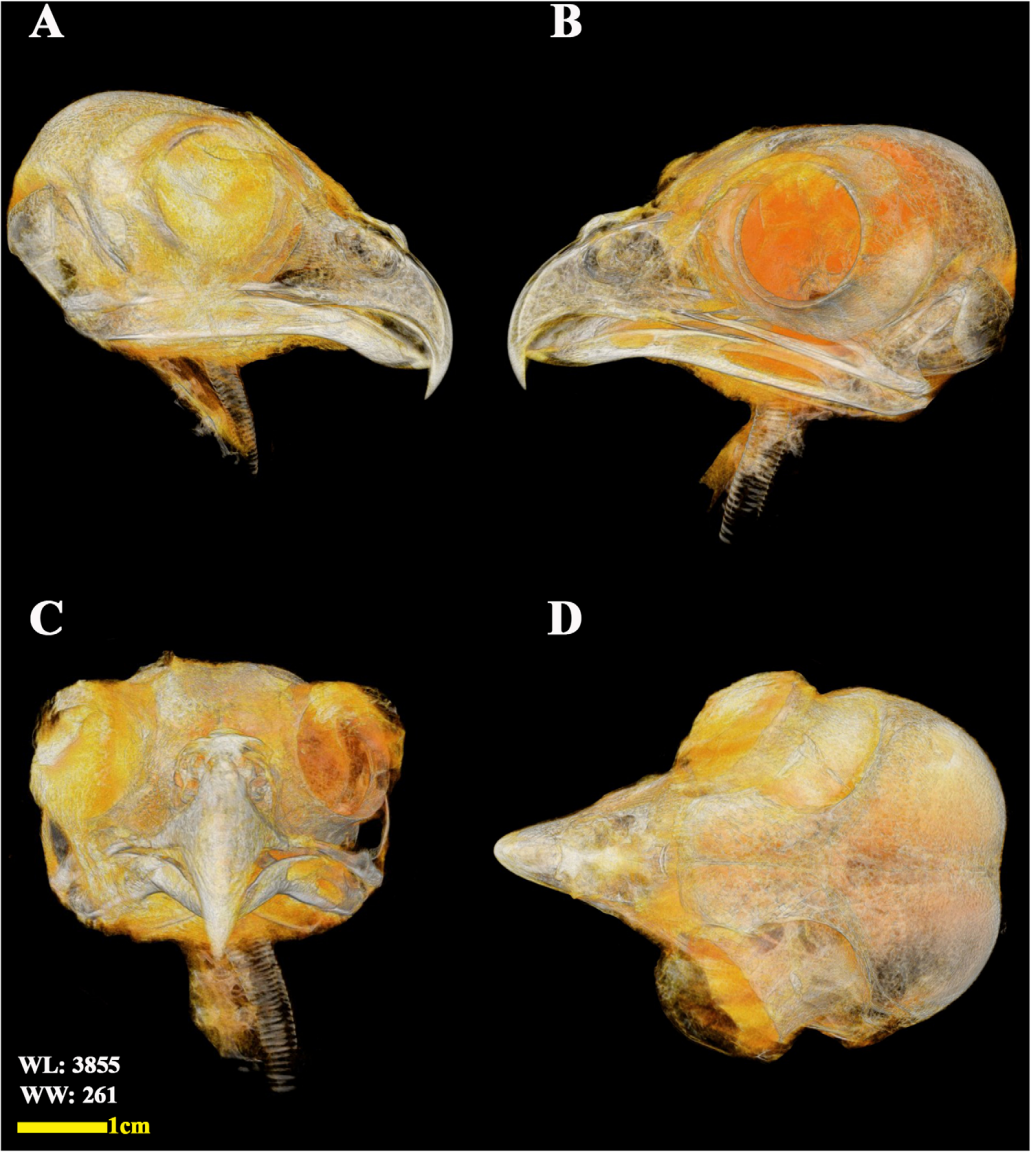
3D reconstructed micro‐CT images of the head region of the little owl (osseous‐shaded‐vp). (A) Right lateral view, (B) left lateral view, (C) anterior view, and (D) dorsal view.

**FIGURE 7:**
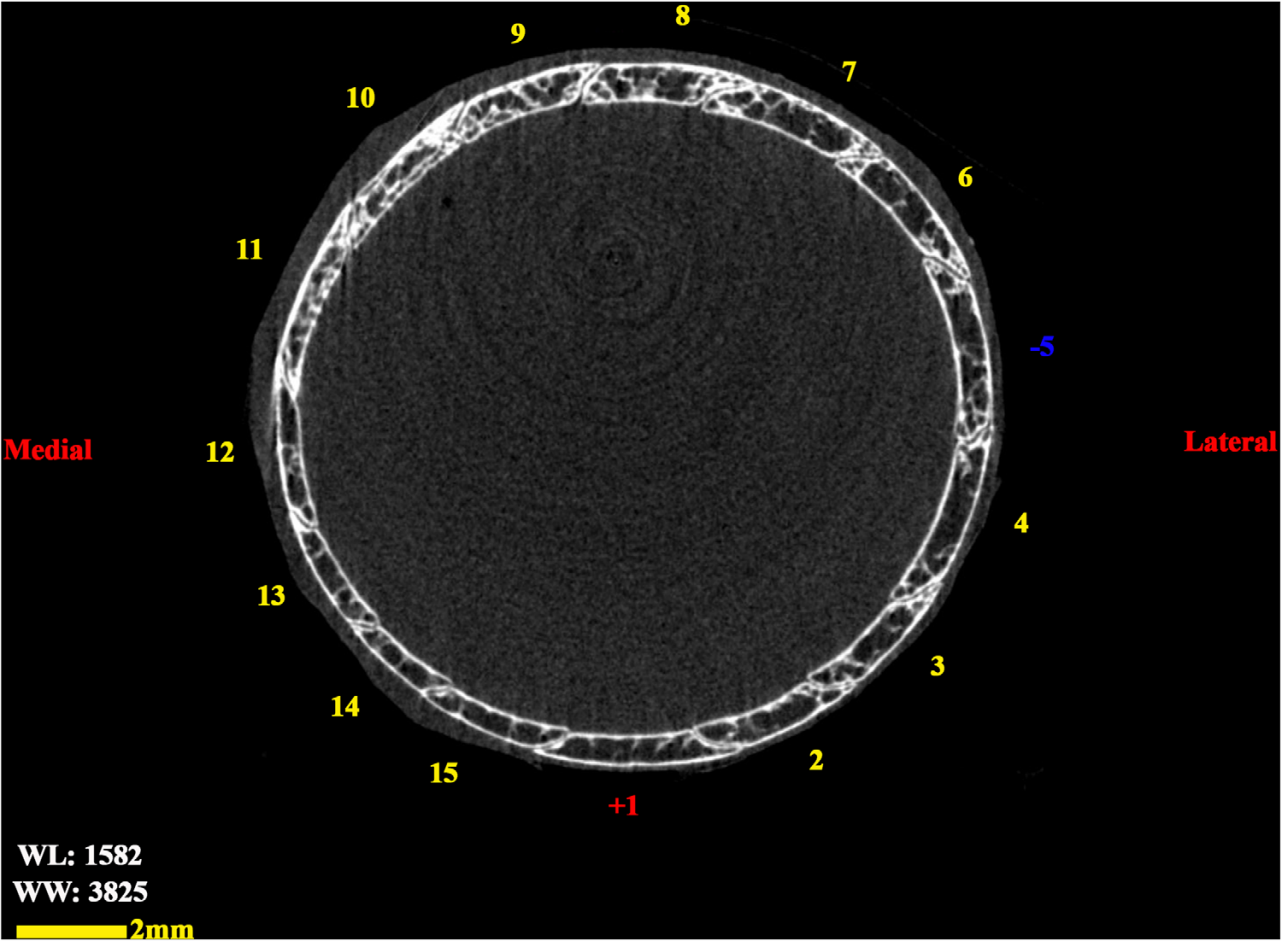
Transverse 2D micro‐CT image of the scleral ring associated with the left ocular structure in a little owl.

**FIGURE 8:**
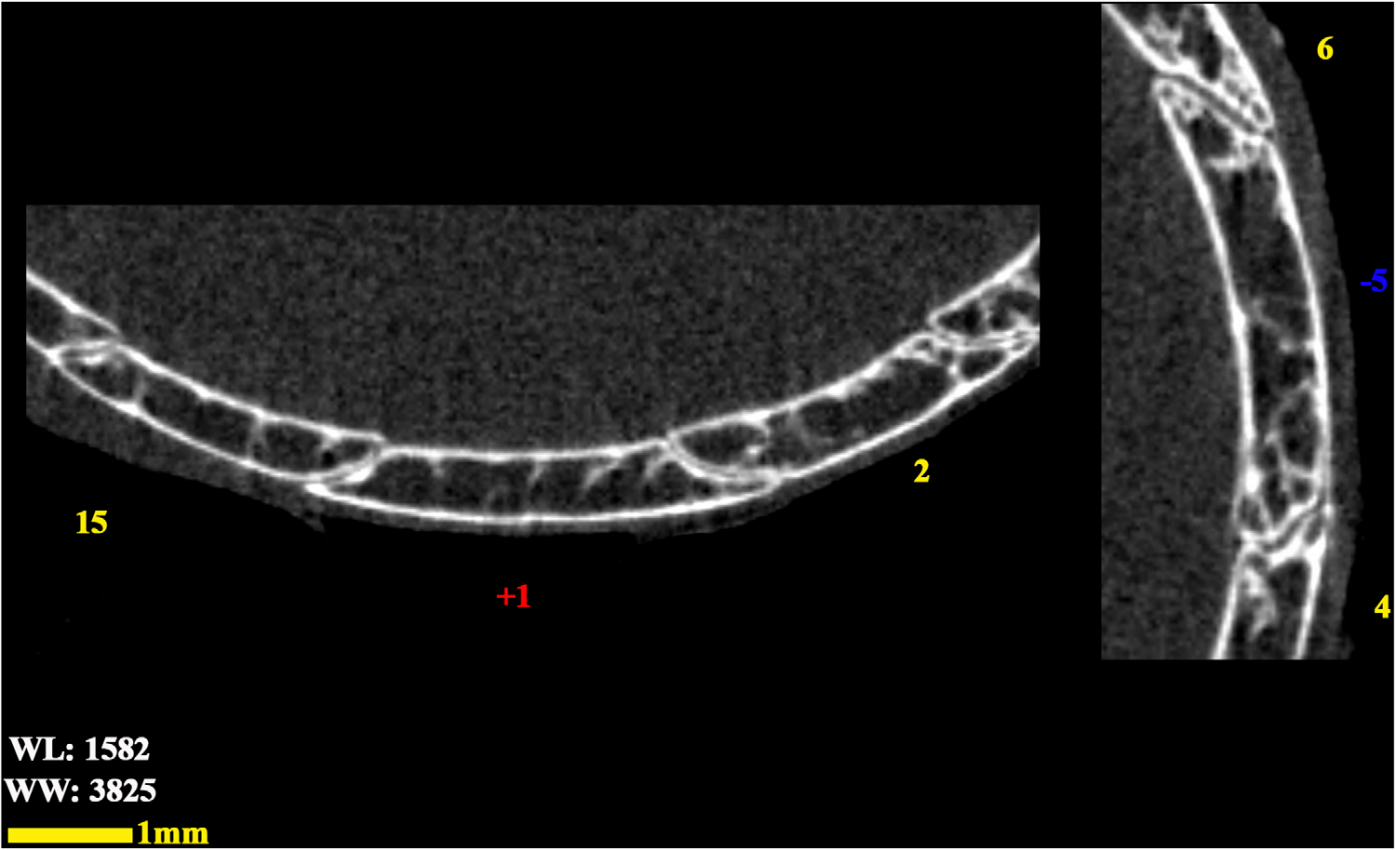
Transverse two‐dimensional micro‐CT scan image (higher magnification of the previous image in the plus and minus ossicles locations), the scleral ring of the left eye in a little owl.

**FIGURE 9:**
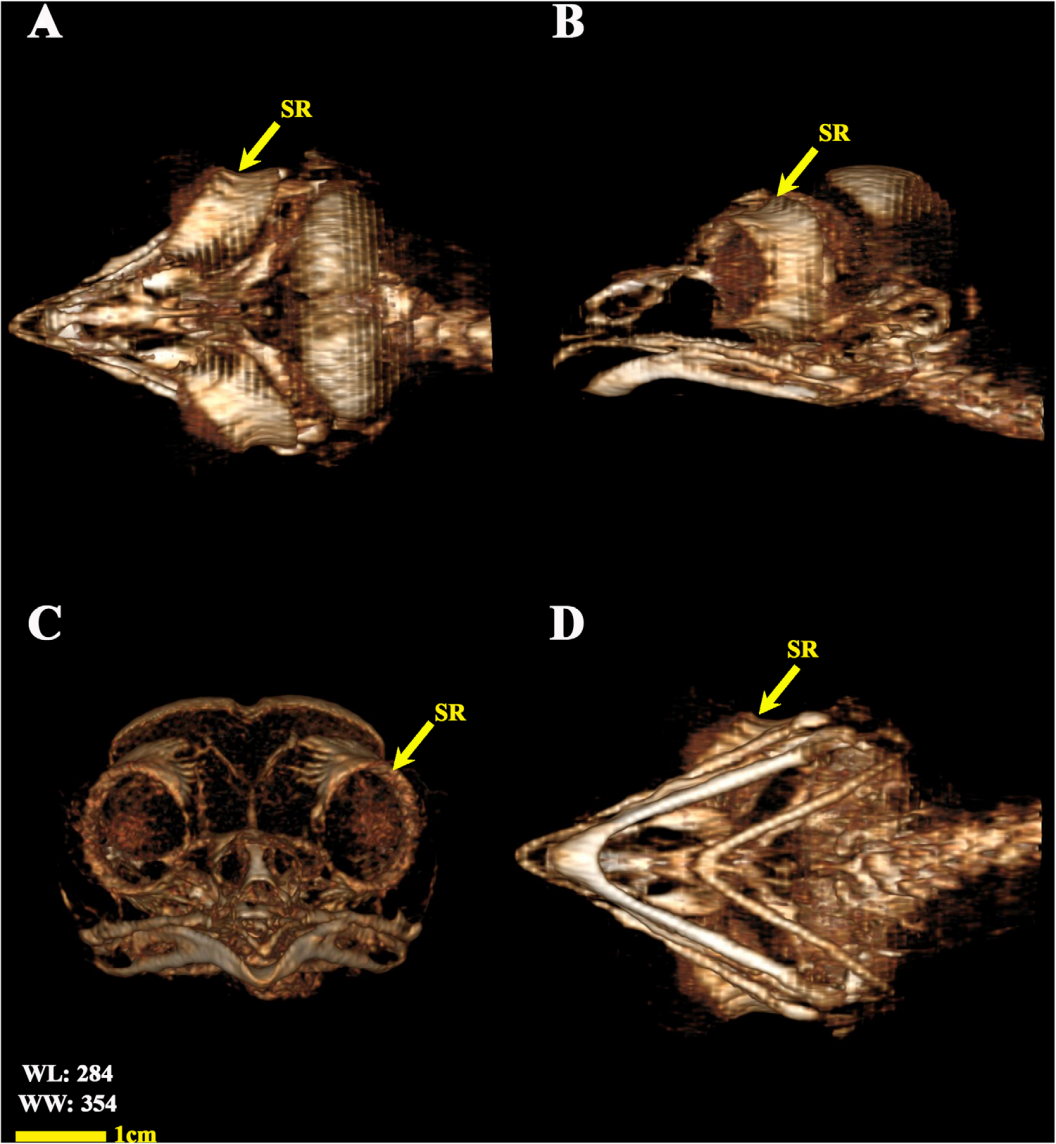
3D reconstructed CT scan images of the little owl's head (osseous‐shaded‐vp). (A) Dorsal view, (B) lateral view, (C) anterior view, and (D) ventral view. SR: scleral ring.

**FIGURE 10:**
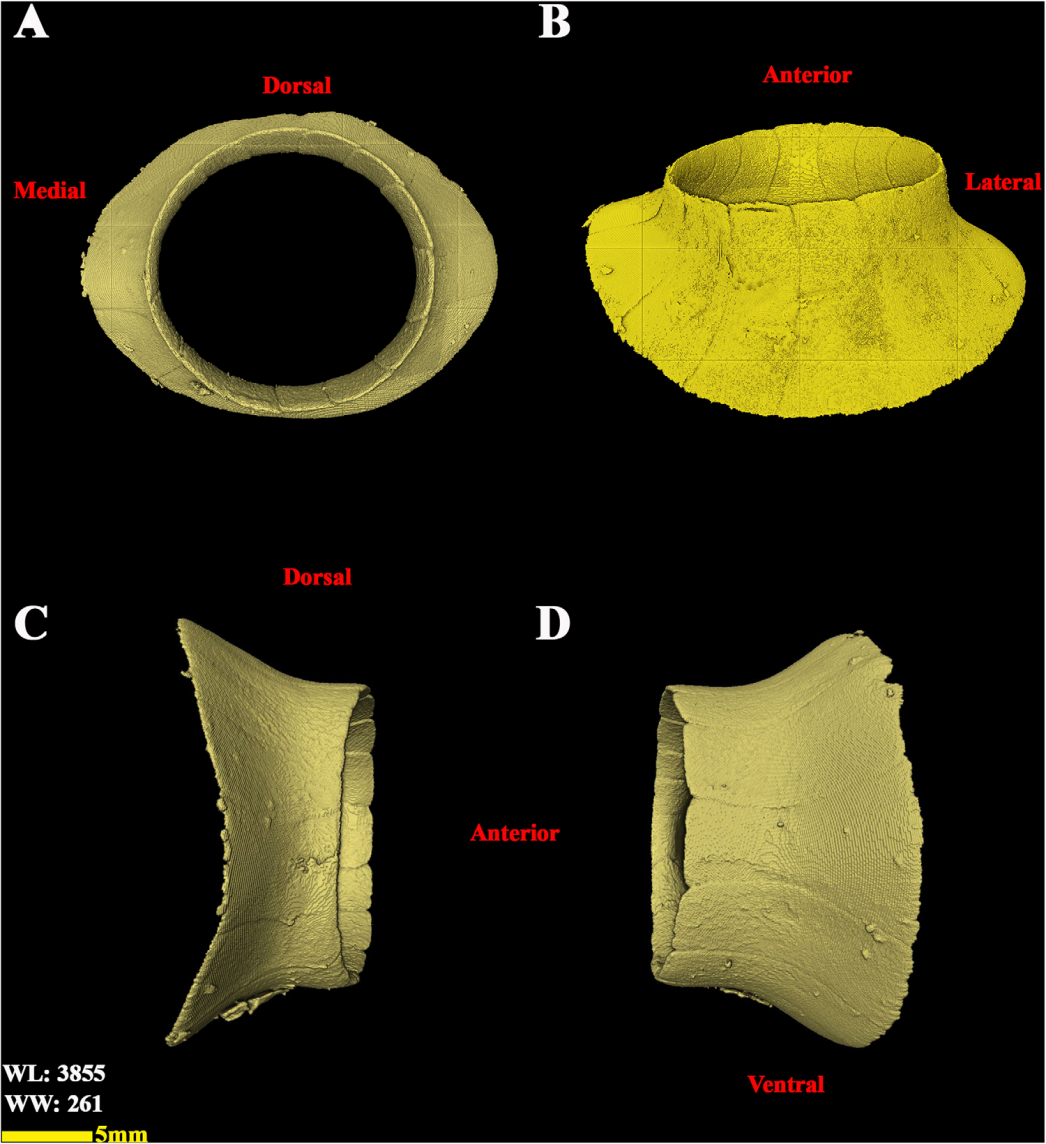
3D reconstructed micro‐CT scan images of the scleral ring of the left eye in a little owl (osseous‐shaded‐vp). (A) Anterior view, (B) ventral view, (C) medial view, and (D) lateral view.

**FIGURE 11:**
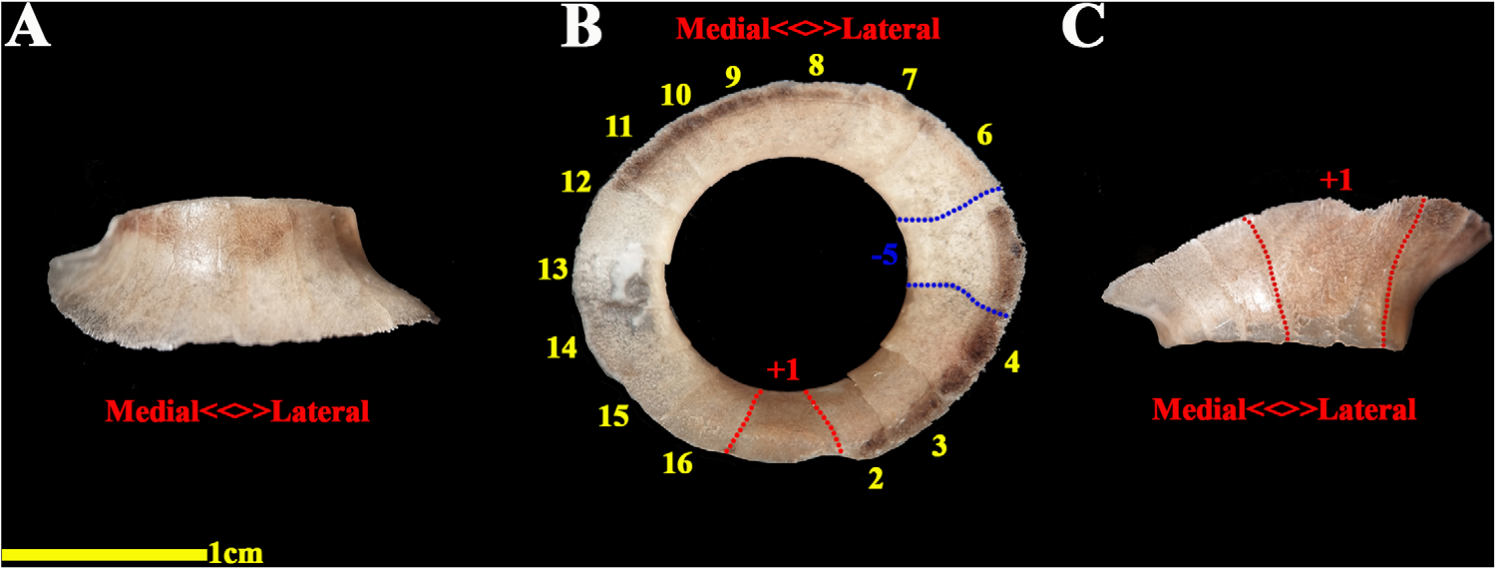
The scleral ring of the left eye in the little owl (*Athene noctua*). (A) Dorsal view, (B) posterior view, and (C) ventral view.

**FIGURE 12:**
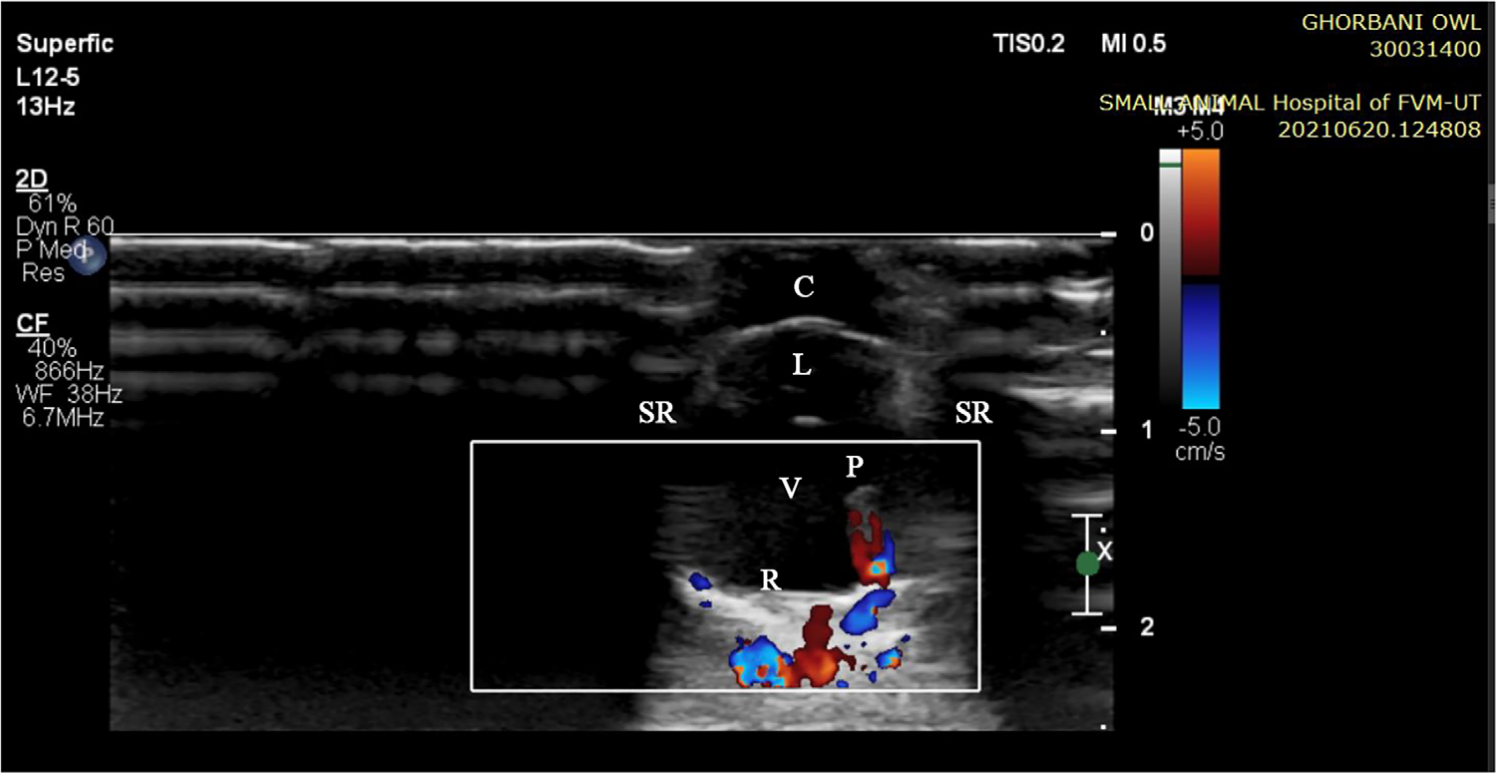
Sagittal Doppler ultrasonogram of the right eye in a little owl. C: anterior and posterior chamber, L: lens, P: *Pecten*, r: retina, SR: acoustic shadow in the region of the scleral ring, V: vitreous chamber.

The analysed specimens exhibited scleral rings with a distinct semi‐hyperbolic morphology, resembling volcanic crater profiles in cross‐section. In the four examined owls, this ring consisted of 15 ossicles and each of these ossicles were quadrangular and rectangular in shape (Figures 3, 4, 5). In an owl, the right scleral ring had 16 ossicles. The placement of the scleral rings in the skull is shown in Figures 2 and 6, and we see the numbering of these ossicles in Figure 7. Among these 15 ossicles, two of them were key ossicles, ossicle number 1 was plus (on the ventral side), and ossicle number 5 was minus and (Figure 8). In a sample of the right eye ring, which had 16 ossicles, ossicle number 1 in the ventral part of the ring was plus ossicle and ossicle number 5 in lateral part of the ring was minus. In this owl, the left ring had 15 ossicles. It should be noted that the sample in which the scleral ring had 16 ossicles belonged to a male owl. These ossicles are concave and their depression is from the middle part of the ossicle to the inside of the scleral ring. The elongated margin of these ossicles was arranged in conjunction with a predetermined angle. The tapered margins of the ossicles formed well‐defined anterior and posterior demarcations of the scleral ring's structural boundaries. The border between these ossicles was somewhat clear in radiographic images, but this border was clearly visible in CT scan images as well as micro‐CT images (Figures 9 and 10). The scleral ring had two general parts: one was the anterior part, whose dorsoventral and mediolateral diameters were apparently equal and gave the appearance of a tube to the anterior part (Figure 11). The other part of the ring was its posterior part, which was almost funnel‐shaped, and its mediolateral diameter was apparently longer than the dorsoventral diameter (Figure 10). In the micro‐CT scan images, the structure of each ossicle was clearly visible, all ossicles had an external cortical part and an internal spongy part (Figure 8), and we can measure each part of it (Table 2). In none of the CT scan and micro‐CT scan images of the samples, no tubercle structure was observed on the ossicles and the sesamoid bone in their vicinity. The anterior edge of the ring fragments was completely smooth, but its posterior edge was completely dentate. During the ocular ultrasound examination, the acoustic shadow observed in Figure 12 corresponds to the high mineral density of the scleral ossicles, which attenuates ultrasound waves.

**TABLE 2:** Spongy or cortical percentage of each ossicle in *Athene noctua*.

Parameter	Spongy	Cortical
Left eye ossicles	47.24 ± 3.29%	52.75 ± 3.29%
Right eye ossicles	51.96 ± 3.76%	48.03 ± 3.76%
All ossicles	49.67 ± 4.22%	50.32 ± 4.22%

### 3.3 Results of Morphological Studies

Tables 3 and 4 show the results of the measurements made in the morphometric study of the head area of owls. Using the independent *t*‐test, a significant difference was seen in the following indicators between male and female owls (*p* < 0.05): left and right anterior diameter, left and right posterior diameter, left and right length of scleral ring, left and right eye ball diameter, left and right optic nerve length, left and right optic nerve sheath diameter, left and right volume of the eyeball, left and right volume of the lens, left and right *Pecten* height, left and right volume of the posterior chamber and anterior chamber, left and right volume of the vitreous chamber, skull length, skull height and skull width (Table 3). Paired *t*‐tests revealed no significant difference between left and right eyes for ossicle thickness (*p* = 0.01), length (*p* = 0.02) or width (*p* = 0.01) (Table 4). The same alphabet in front of the parameters means that there is no significant difference between them.

**TABLE 3:** Indicators measured by gender in *Athene noctua*.

Index	Group	Mean (95% CI)	SEM
Left anterior diameter (cm)	Male	1.53 a (1.16–1.90)	0.13
	Female	2.27 b (1.91–2.64)	0.13
Right anterior diameter (cm)	Male	1.50 a (1.14–1.87)	0.13
	Female	2.25 b (1.89–2.62)	0.13
Left posterior diameter (cm)	Male	2.35 c (1.99–2.72)	0.13
	Female	3.10 d (2.74–3.47)	0.13
Right posterior diameter (cm)	Male	2.63 c (2.26–3.00)	0.13
	Female	3.37 d (3.01–3.74)	0.13
Left length of scleral ring (cm)	Male	1.05 e (0.69–1.42)	0.13
	Female	1.80 f (1.44–2.17)	0.13
Right length of scleral ring (cm)	Male	1.13 e (0.77–1.50)	0.13
	Female	1.89 f (1.52–2.25)	0.13
Left eye ball diameter (cm)	Male	2.43 g (2.07–2.80)	0.13
	Female	3.18 h (2.82–3.55)	0.13
Right eye ball diameter (cm)	Male	2.44 g (2.08–2.81)	0.13
	Female	3.19 h (2.83–3.56)	0.13
Left optic nerve length (cm)	Male	0.50 i (0.14–0.87)	0.13
	Female	1.25 j (0.89–1.62)	0.13
Right optic nerve length (cm)	Male	0.50 i (0.10–0.83)	0.13
	Female	1.25 j (0.89–1.62)	0.13
Left optic nerve sheath diameter (cm)	Male	0.50 k (0.89–1.62)	0.15
	Female	1.32 l (1.08–1.56)	0.12
Right optic nerve sheath diameter (cm)	Male	0.45 k (0.09–0.82)	0.13
	Female	1.20 l (0.84–1.57)	0.13
Left volume of the eyeball (cm^3^)	Male	4.87 m (4.51–5.24)	0.13
	Female	5.62 n (5.26–5.99)	0.13
Right volume of the eyeball (cm^3^)	Male	5.00 m (4.64–5.37)	0.13
	Female	5.75 n (5.39–6.12)	0.13
Left volume of the lens (cm^3^)	Male	1.11 o (0.75–1.48)	0.13
	Female	1.86 p (1.50–2.23)	0.13
Right volume of the lens (cm^3^)	Male	1.09 o (0.73–1.46)	0.13
	Female	1.85 p (1.48–2.21)	0.13
Left *Pecten* height (mm)	Male	5.27 q (4.91–5.64)	0.13
	Female	6.15 r (5.79–6.52)	0.13
Right *Pecten* height (mm)	Male	5.19 q (4.83–5.54)	0.13
	Female	6.07 r (5.71–6.44)	0.13
Left volume of anterior and posterior chamber (cm^3^)	Male	1.14 s (0.77–1.50)	0.13
	Female	1.90 t (1.53–2.27)	0.13
Right volume of anterior and posterior chamber (cm^3^)	Male	1.12 s (0.74–1.51)	0.14
	Female	1.86 t (1.50–2.23)	0.13
Left volume of the vitreous chamber (cm^3^)	Male	3.37 u (3.01–3.74)	0.13
	Female	4.13 v (3.76–4.49)	0.13
Right volume of the vitreous chamber (cm^3^)	Male	3.34 u (2.97–3.70)	0.13
	Female	4.09 v (3.72–4.46)	0.13
Skull length (cm)	Male	5.72 w (5.45–5.99)	0.14
	Female	6.54 x (6.29–6.79)	0.13
Skull height (cm)	Male	3.74 y (3.49–3.99)	0.13
	Female	4.55 z (4.30–4.80)	0.13
Skull width (cm)	Male	4.22 α (3.95–4.49)	0.14
	Female	4.56 β (4.31–4.81)	0.13
Volume of the brain (cm^3^)	Male	8.76 ɤ (8.47–9.05)	0.15
	Female	9.97 Ω (9.75–10.19)	0.11

**TABLE 4:** The size of the ossicles in *Athene noctua*.

Parameter	Mean ± SEM (95% CI) of left eye	Mean ± SEM (95% CI) of right eye
The thickness of each ossicle (mm)	0.55 ± 0.09 a (0.37–0.73)	0.58 ± 0.08 a (0.42–0.74)
The length of each ossicle (mm)	6.39 ± 1.07 b (4.29–8.49)	6 ± 0.96 b (4.12–7.88)
The width of each ossicle (mm)	3.69 ± 0.78 c (2.16–5.22)	3.03 ± 0.59 c (1.87–4.19)

## 4 Discussion

While scleral ring morphology contributes to taxonomic identification, it should be combined with other morphological and molecular traits (Fischer and Schoenemann 2019). In the order of owls, falcons and water birds, the shape of the eyeball affects the shape of its ossicles, so that the eyeball becomes tubular, and for this reason, the shape of the eyeball does not change due to air and water pressure (Tidwell 1999). It should be mentioned that bone structures in the eye prevent the passage of ultrasonic waves, and this is very important in ultrasound examinations (King and McLelland 1984).

Thus far, a limited number of investigations have been conducted regarding the ocular globe of *A. noctua*, and the scleral ring has not been examined in any of these inquiries. In prior research, the morphology of the scleral ring in various avian species, including other owls and penguins, has been analysed; however, the scleral ossicles have not been quantified in any of these studies. In the present investigation, the anatomy of the ocular globe, scleral ring and ossicles has been meticulously examined.

There exist documented instances regarding the fracture of the scleral ring in various avian species as a consequence of cranial trauma, and its subsequent diagnostic approaches; Lindley et al. (1988) detailed the identification of a fracture in the scleral ring of *Buteo jamaicensis* utilizing radiographic techniques (König et al. 2016). In the year 1987, researchers conducted an examination of the structural attributes of the scleral ring alongside its phylogenetic affiliations in *Ophisthocomus hoazin* (Franz‐Odendaal and Vickaryous 2006).

In an investigation focused on the evolutionary lineage of the scleral ring in both fish and avian species, it has been emphasised that during embryogenesis, the osseous components constituting this anatomical feature arise from scleral cartilage, whereas in avians, these are classified as dermal bones (Lima et al. 2017). In 2012, Zhang et al. (2012) examined the morphology of ossicles and their ossification during growth stages in chicken. The structure of ossicles in Brazilian birds has been investigated in 2009. In this study, it is mentioned that the number of ossicles in each eye of *Athene cunicularia* is 17 (Lima et al. 2017). In another study in 2006, they investigated some characteristics of healthy eyeballs in all kinds of birds, such as the height of the *Pecten*, and some of the abnormalities that were detected were reported, although the gender and weight of the birds were not mentioned in this article (Gumpenberger and Kolm 2006).

In Bohórquez Mahecha and de Oliveira (1998), investigations revealed that the scleral ring present in owls, as well as certain avian species, contains a previously unobserved and undocumented osseous structure; this bone is situated along the trajectory of the pyramidal muscle tendon and features a channel for its passage. In this study, as mentioned, no sesamoid bone structure was observed in the vicinity of the ring, and no groove was observed on the external surface of the ring. It has been documented that the anatomical configuration of the scleral sesamoid bone was not discerned in *Glaucidium brasilianum*, but in *A. cunicularia*, it is a round and small structure, in *Bubo bubo*, it is an elongated structure, and in *Bubo virginianus* it is an elongated and two‐piece structure (Lima et al. 2017). *A. otus* is one of the owls on which diagnostic and anatomical imaging studies have been performed on its head and eye area. The new scientific name of this species is *Megascops asio*, and this species belongs to North America (Morgan et al. 1994).

The unilateral presence of 16 ossicles in one specimen represents a notable anatomical variation. Such asymmetry in ossicle count, while rare, has been documented in other Strigiformes and may arise from subtle variations during embryonic development of the scleral cartilages. Future studies with larger sample sizes are needed to determine the prevalence and potential functional implications of this variation.

In another study, the characteristics of the eyeballs of a variety of bird species, including the *A. otus*, were measured. In that study, five cases of *A. otus* were studied, although in that study, comparisons between male and female sexes and right and left eyeballs were not done, which were investigated in this study (Gumpenberger and Kolm 2006).

In the investigations undertaken regarding the ocular anatomy of the horned owl by Morgan et al. (1994), it is elucidated that the scleral ring of *A. otus* comprises two distinct components, namely anterior and posterior segments, with the anterior segment exhibiting a tubular configuration characterised by an almost circular cross‐section, while the posterior segment displays a funnel‐like morphology with an approximately oval cross‐section. The ocular lens is situated adjacent to the anterior segment, and on the ventromedial aspect of the scleral ring, numerous small tubercles can be observed on its external surface (Morgan et al. 1994). Concerning the scleral rings of both the left and right eyes, it can be asserted that there exists no discernible variation between them in terms of the number of ossicles, their morphology, or dimensions, thus indicating bilateral symmetry. The ocular structures, along with the majority of related anatomical features scrutinised in this research, were observed to be significantly larger in females compared to males, with similar to other ocular parameters, *Pecten* height was significantly greater in females compared to males (Table 3), which is likely an allometric consequence of their larger overall body and eye size. The proportion of the aggregate volume of the right and left ocular structures to the volume of the brain is quantified as 2.42 in the male *A. otus* and 2.31 in the female. This number is 1.07 in the male and 1.14 in the female in the study conducted on the little owl (Table 5), and we come to the conclusion that in the little owl, the ratio of the volume of the eyes to the brain is smaller than that of the horned owl (Zehtabvar et al. 2022).

**TABLE 5:** Ratios of total eye volume to brain volume in male and female *Athene noctua*.

Sample number	Male owls	Female owls
1	1.04	1.11
2	1.08	1.14
3	1.12	1.17
4	1.04	1.13
5	1.10	1.16

Mean	1.07 a	1.14 a
SEM	0.02	0.01

According to the studies on the scleral ring of different penguin species using micro‐CT scan, in *Pygoscelis papua* chicks (at the age of 10 weeks), the amount of spongy bone tissue in the ossicles is much higher than in adults and there are many empty spaces in chicks. It is seen between bone tissues (Hadden et al. 2021). In our study, which was conducted on an adult owl, about 50% of the volume of each ossicle is cortical bone and the remaining 50% is spongy bone (Table 2).

In four specimens of the scleral ring of *Guttera plumifera* that were scrutinised, it was noted that all specimens possess 14 ossicles. In three specimens, the scleral ring was classified as Type A and exhibited four key ossicles, whereas in one ring, merely two key ossicles were observed, and it was classified as Type B. This investigation demonstrated that the classification of the scleral ring may vary within the same species; however, in our examination, all specimens were consistent regarding the quantity of key ossicles (Queiroz and Good 1988).

The use of ketamine and xylazine anaesthesia is known to potentially affect intraocular pressure and globe dimensions. While every effort was made to perform measurements consistently post‐induction, this represents a potential limitation of our in vivo data that should be considered when interpreting the results, particularly for chamber volumes.

In a study conducted on *Pecten* in *Coturnix japonica* species, it is stated that the structure of *Pecten* is responsible for light absorption and temperature regulation. *Pecten* is a structure that has many vessels and pigment, originates from the retina and is located inside the vitreous. The scleral ring displays isometric proportions between sexes in Japanese quail, showing no gender‐based size variation. It was observed in the little owl that the height of the *Pecten* in the female is higher than that of the male; of course, it should be noted that the female is generally larger than the male, and the different morphometric parameters of the female are also larger than the male (Orhan et al. 2011).

Microstructural analysis revealed significant differences in scleral ossicle composition between species. In *Struthio camelus*, ossicles demonstrated a cortical‐to‐trabecular bone ratio of approximately 2:1 (67% cortical vs. 33% trabecular), while *A. noctua* exhibited a balanced 1:1 ratio (50% cortical vs. 50% trabecular). The ostrich scleral ring contained 15–17 ossicles with four ‘excellent’ ossicles (Type A configuration), contrasting with the little owl's consistent 15‐ossicle arrangement featuring only two key ossicles (Type B). Furthermore, the owl's ring displayed a characteristic tubular morphology absent in the non‐tubular ostrich ring (Masoudifard et al. 2025).

## 5 Conclusion

In all bone samples isolated in this study, it was observed that there are two key ossicles in each eye, one of which is plus and one is minus, and the rest of the ossicles are interlocking. Therefore, the scleral ring observed in the *A. noctua* exhibits characteristics of Type B, analogous to the examined species of owls,

The scleral ring in the *A. noctua* exhibits two distinct components, defined as anterior and posterior sections, with the anterior section characterised by a tubular morphology exhibiting an almost circular cross‐section, while the posterior section assumes a funnel‐like configuration with a nearly oval cross‐section. Positioned immediately posterior to the anterior segment, the ocular lens establishes a direct anatomical continuity with anterior chamber structures. It is noteworthy that the scleral ring in this particular species is devoid of tubercular and sesamoid bone structures. In certain species of owls, a disparity in the number of scleral ring ossicles between the right and left eyes may be observed. The ocular structures and their associated components analysed in this investigation demonstrated a greater size in females compared to males. These observations, along with their implications for the visual capabilities of both sexes, warrant further consideration and exploration in subsequent research endeavours.

## Author Contributions

**Omid Zehtabvar**: conceptualization, investigation, funding acquisition, writing – original draft, writing – review and editing, methodology, validation. **Majid Masoudifard**: investigation, methodology, funding acquisition. **Hesameddin Akbarein**: investigation, methodology, funding acquisition. **Arman Shahbazi**: review and editing. **Setayesh Karimi**: review and editing. **Sara Binesh**: review and editing. **Elnaz Khalilzadeh**: review and editing.

## Funding

The authors have nothing to report.

## Ethics Statement

This study was a DVM thesis, and all experimental procedures were approved by the Faculty of Veterinary Medicine, University of Tehran (30704/6/10).

## Conflicts of Interest

The authors declare no conflicts of interest.

## Data Availability

Data are available on request from the authors.
